# Conference Report: COMPASS ’96 THE ELEVENTH ANNUAL CONFERENCE ON COMPUTER ASSURANCE Gaithersburg, MD June 17–21, 1996

**DOI:** 10.6028/jres.101.075

**Published:** 1996

**Authors:** Karen Ferraiolo, Laura M. Ippolito

**Affiliations:** Arca Systems, Inc., Columbia, MD 21044; Computer Systems Laboratory, National Institute of Standards and Technology, Gaithersburg, MD 20899-0001

## 1. Introduction

The Eleventh Annual Conference on Computer Assurance (COMPASS ’96) was hosted by the Computer Systems Laboratory of the National Institute of Standards and Technology on June 17–21, 1996. COMPASS is an organization whose mission is to advance the theory and practice of building assurance into critical computer systems. Each year, COMPASS brings together researchers, developers, and evaluators of high assurance computer systems with the goal of bridging the gap between research and practice. The annual conferences are cosponsored by the Institute of Electrical and Electronics Engineers, Inc. (IEEE) Aerospace and Electronics Systems Society and the IEEE National Capital Area Council. COMPASS ‘96 industry, government, and academic cosponsors included: Arca Systems, Inc.; BDM; Computer Associates; Food & Drug Administration; George Mason University; Intermetrics; Logicon, Inc.; Kaman Sciences Corporation; MITRE; NIST; Naval Research Laboratory (NRL); Reliable Software Technologies (RST) Corporation; Space and Naval Warfare Systems Command; Systems Safety Society; TRW Government Information Services Division; and Trusted Information Systems.

COMPASS ’96, with its theme of “Industrial Strength Research,” consisted of paper presentations, panels, a banquet speaker (Nancy Leveson, University of Washington), and a tools fair which addressed the development of new technology, the application of technology, and the transfer of research into practice. Specific topics included formal methods, verification, testing, safety, and security. This year’s conference attracted over 120 participants from the U.S. government, industry, and academia and included representation from foreign countries such as Canada, Denmark, Germany, Israel, Italy, the Netherlands, Sweden, and the United Kingdom (U.K.).

## 2. Tutorials

Two tutorial tracks on Monday and Tuesday provided attendees with information on safety and assurance concepts, use of formal tools and analysis, and human-machine interface concerns. For Monday’s full-day tutorial, Dr. John McDermid from the University of York, U.K., described “Safety Case Construction and Management.” The development of safety cases in the U.K. is similar to the safety certification processes performed in the United States. Dr. McDermid focused on the need to clearly present high level arguments (HLA) along with the detailed supporting evidence. He remarked that the HLAs are often lacking in safety cases that are frequently filled with large amounts of evidence details, presenting major challenges to the reviewers. The tutorial touched upon safety engineering techniques and methods, with illustrations of specific safety case examples and on-line tool support.

Michael Harrison of the University of York, U.K., presented the first half-day tutorial “Impact and Design of the Human-Machine Interface.” Using examples, the tutorial demonstrated the need for systems to meet ease-of-use as well as functional requirements. The tutorial described how human error tolerance requirements may be derived in a form that can be used to specify system behavior.

A second half-day tutorial on “Automatic Formal Analysis of Cryptographic Protocols” featured Dr. Steve Brackin of Arca Systems, Inc. The tutorial illustrated the use of software tools to automatically perform belief-logic authentication analyses of cryptographic protocols. The tutorial included: an overview of cryptographic protocols, protocol failure, and belief-logic; a description of the interface specification language (ISL) used to specify protocol properties; and demonstrations of the use of ISL and supporting tools for analyzing cryptographic protocols.

On Tuesday, J. Strother Moore and William D. Young of Computational Logic gave a full-day tutorial on “ACL2.” ACL2 is an extended, reimplemented version of the Boyer-Moore Nqthm theorem prover that supports the applicative subset of Common Lisp as its logic. Using examples, the tutorial provided an understanding of the ACL2 logic and the theorem prover.

Douglas Landoll of Arca Systems, Inc., presented a half-day tutorial “A Framework for Reasoning about Assurance.” This tutorial explored assurance by describing a framework for reasoning about the methods, artifacts, and concepts involved with producing and analyzing assurance. Mr. Landoll described how, given various types of evidence, the framework can be used to develop concise and complete assurance arguments. The framework is documented in a report to be published by NIST.

Albert M. K. Cheng of the University of Houston gave a half-day tutorial on “Formal Analysis and Verification of Real-Time Systems.” Mr. Cheng presented the basics of the technology for building next generation real-time systems capable of performing complex monitoring and control functions while meeting timing constraints.

## 3. General Conference

Karen Ferraiolo, General Chair, opened COMPASS ’96 with welcoming remarks. Connie Heitmeyer and Stuart Faulk, Program Chairs, discussed the program and invited attendees to participate by sharing their ideas and experiences. Andrew Moore, of the Tools Fair Committee, presented the tools available for demonstration including: *SafeNet: Software Safety Analysis Tool* by RST Corporation; *Tablewise, Z/EVES*, and *VHDL* by ORA; SCR*: Toolset for Specifying and Analyzing Requirements by the NRL; *QuickCheck: Model Checking Software Requirements* by the University of Waterloo; *Foresight: Requirements Traceability and Management* by Nu-Thena Systems; *IV&V Integrated Support Environment* by Intermetrics; *ObjecTime: An Object-Oriented Toolset* by Objectime; and, *KIDS and Specware: Programming Environments* by Kestrel and the National Security Agency.

Two keynote speakers from industry described successful efforts in transferring new software technology to commercial high assurance systems. On Wednesday, David Weiss from Lucent Technologies Bell Laboratories spoke on “Family-Oriented Abstraction, Specification and Translation: The FAST Process—A Study in Successful Technology Transfer.” He stated that in order for technology transfer to be successful, there must be immediate benefit and long term benefits. Defining a family as a set of programs, he described FAST as the process for defining families and developing environments for generating family members with the goals of changing the current way of developing software and taking better advantage of existing knowledge. The approach was to integrate the development process and the product (e.g., concurrent engineering) and to reorganize the software development process to evolve a family rather than build single systems. Key to improvement is the reorganization of software production to take advantage of the family viewpoint with one organization focused on continually improving production of family members (process oriented) and one organization that determined the requirements for family members (project oriented).

Mike Viola of Ontario Hydro, Canada, opened the conference on Thursday with his keynote, “Ontario Hydro’s Experience with New Methods for Engineering Safety-Critical Software.” Use of digital systems in safety systems led to regulatory problems because of lack of widely accepted definition of “safe enough.” This led Ontario Hydro and AECL to develop a series of standards and procedures based on past experiences. The new methods include mathematically precise specification of requirements and design, systematic design and code verification, software hazard analysis and software reliability demonstration. These new methods resulted in cost-effectiveness, added more rigorous verification, and avoided regulatory paralysis. Standards, procedures and tools are being further refined to reflect this experience.

## 4. Formal Specification and Verification I

John Gannon from the University of Maryland moderated the first session of the conference. H. Shen of McMaster University, Canada, presented the first paper, “Table Transformation Tools: Why and How” (coauthored by J. Zucker and D. L. Parnas, McMaster University). He described a prototype table transformation tool for inverting tabular representations. He discussed tabular formats of widely varying characteristics, since a tabular form suitable for one application may not be suitable for another. He referred to algorithms that are used for transforming one tabular format to another. One could choose a tabular format that is most suitable for that application for software documentation. The tabular documentation has the advantages of being simple and precise; it is in a format that can be analyzed systematically to be consistent and complete.

Douglas Stuart, University of Texas at Austin, spoke on “Simulation vs Verification: Getting the Best of Both Worlds” (coauthored by Aloyusius K. Mok, University of Texas). He described a technique of combining simulation and verification to analyze real-time system specifications. A tool XSVT has been implemented using MT toolset for ModeChart. This method can be used for focused verification of critical sections of the requirements by generating a computation graph fragment and stopping when a user defined frontier is reached.

## 5. Mechanical Theorem Provers

Ralph Jeffords from NRL moderated the session on theorem provers. J. Strother Moore of Computational Logic, Inc. presented the first paper on “ACL2: An Industrial Strength Version of NQTHM” (coauthored by Matt Kaufmann, Computational Logic, Inc.). Mr. Moore talked about several applications that used ACL2. Examples included the AMD5k86 processor floating point division algorithm, Berkeley C String Mover, Motorola Complex Arithmetic Processor, and a Finite Input Response filter. ACL2 allows rapid prototyping of models of systems, efficient execution of those models, and proofs of deep theorems about those models, but can be labor-intensive.

William Young of Computational Logic, Inc., author of “Interactive Consistency in ACL2,” described interactive consistency conditions, that is, a collection of processors communicating only via message passing arriving at a consensus view of some common state with some processor possibly faulty. As an example, he used the Oral Message algorithm, sometimes called the “Byzantine Generals Problem.” ACL2 was used to analyze both a symmetric version and an asymmetric version of the algorithm. It was concluded that different theorem provers are suited to different classes of problems and that comparisons between provers are easy, but wrong conclusions can be drawn. Also, with the current state of the art of theorem provers, the tools are hard to use and can be used only by those with background and experience in their application.

Sakthi Subramanian of Trusted Information Systems, presenting “Mechanical Verification of Object Code vs. Source Code,” (coauthor Jeffrey Cook, Trusted Information Systems) examined the problem of verifying that compiled C code implements the corresponding C source code. He described an object code verification project to verify each object code program individually without verifying the compiler. He gave steps to the approach, example states, example lemmas, and steps carried out by the tool. Two short C programs were given as examples. Conclusions showed that automatic verification of straight line programs in C or JAVA appear to be a feasible first step for industrial application of formal methods. It can possibly be done on Ada programs, even though it was not tried as part of the current effort.

## 6. Practical Applications of Formal Methods

Stefan Wittmann of Bundesamt fur Sicherheit in der Informationstechnik, Germany, presented the paper “Industrial Usage of Formal Development Methods: The VSE-Tool Applied in Pilot Projects” (coauthored by Frank Koob and Markus Ullmann, Bundesamt fur Sicherheit in der Informationstechnik.) He described Verification Support Environment, a verification tool that allows use of formal and semi-formal development methods combined with any work environment, so that engineers and developers do not have to give up their usual work environment in order to use a formal method.

Dino Mandrioli (Politecnico di Milano, Italy) presented “Specifying, Validating, and Testing a Traffic Management System in the TRIO Environment” (authored by Angelo Gargantini, Lilia Liberati, and Angelo Morzenti of Politecnico di Milano and Cristiano Zacchetti of ATM-Azienda Transporti Municipale, Italy). He reported on the group’s experience in applying a formal method to the specification and design of a system for monitoring and controlling surface vehicle traffic in a densely populated urban area. The software engineering environment was based on TRIO, a linear time metric temporal logic. Several validation inaccuracies were found, although no significant verification errors were found. Using TRIO instead of a more traditional development method led to a shift in resource allocation from the design and acceptance stages to the requirements and validation stages, significantly reducing overall costs.

Joanne M. Atlee of University of Waterloo, Canada, presented “Feasibility of Model Checking Software Requirements: A Case Study” (coauthored by Tirumale Sreemani, University of Waterloo). She outlined a case study that demonstrated the feasibility of symbolic model checking to determine if a property is a theorem of a given specification. The software requirements of the A-7E aircraft were analyzed using McMillan’s Symbolic Model Verifier.

## 7. Panel: High Assurance Computing

Panel Moderator, Rich Gerber (University of Maryland), introduced the topic: “Assuming we have sufficient software infrastructure to support systems that are reasonably secure, fault tolerant, meet safety criteria, and adhere to fault tolerant properties, we still cannot integrate current methods to satisfy a combination of these properties, nor find a way to scale up to a very large system.”

John Knight, University of Virginia, considered scaling up to be the mega issue. He feels we are not very far along in our long track to achieve high assurance in all arenas: security, fault tolerance, safety, and real time. Ricky Butler, NASA Langley Research Center, believes we are on the right track if we mean the research community and focused industry. He suggested that the management of software processes is good, but software engineers need to be able to engineer, design, and develop well. Dr. Butler illustrated the components of the “right track,” and made the observation that academic successes are not taken up by industry; they are left as prototypes. Nancy Leveson, University of Washington, asserted that the primary impediment is sociological rather than technical. She believes safety is a hard sell in the market place. We have the right ideas, but not enough people have been killed yet. The primary impediment is “not seeing the need until there is a major disaster.”

John McLean, NRL, presented his ideas through a security perspective. He discussed the historical approaches to confidentiality and today’s broader security challenges. He remarked that we can’t really define deterministic models. We need a technology to integrate probabilistic properties when one type may undercut another (e.g., security and real time). We really need predictability. We have a difficult enough time with individual critical properties; when we combine them we are worse off. Dick Kemmerer, University of California, Santa Barbara, discussed what distinguishes high assurance systems. He stated that we are really talking about critical systems with a “feel” that they work properly. He questioned when we should view critical properties as orthogonal and when we should view them simultaneously. An observation was made about conversations between real-time engineers and security engineers; it presented a startling recognition of totally different goals.

## 8. Program Verification

Connie Heitmeyer of NRL moderated this session. Anne Elizabeth Haxthausen of the Technical University of Denmark presented her paper “Developing a Translator from C programs to Data Flow Graphs using RAISE.” She discussed the use of the Rigorous Approach to Industrial Software Engineering (RAISE) method to develop a translator which would translate C programs to data flow graphs, and described the RAISE Specification Language, purpose of the translator, and the process of translation. She stated that while the specification is formal, verification may be informal. Examples of simple code segments were given. Several conclusions were drawn, including that while it can increase the reliability, it takes time to learn, and that the process can be used with Ada but a different translator is needed.

Marsha Chechik of the University of Maryland presented a paper as part of her Ph.D. work: “Verification of Consistency Between Concurrent Program Designs and their Requirements” (coauthored by John Gannon, University of Maryland). The goal of the project was to develop an aid in the acceptance and use of precise documentation of requirements, and to amortize the creation costs. The Software Cost Reduction requirements, the Program Design Language used, verification of properties (including both pessimistic and optimistic properties), and the development of the model were described. An example of a Mutual Exclusion System, including the building of a concurrent design flow graph, was used to illustrate the process. An example illustrated its ability to find errors.

## 9. Formal Specification and Verification II

This session was moderated by Dino Mandrioli, Politecnico di Milano, Italy. Ashvin Dsouza of Cornell University presented “Verifying SOS Specifications” (coauthored by Bard Bloom and Allan Cheng, Cornell University). He talked about a process algebra-like specification language designed to specify processes and protocols by which they interact. A family of specification languages (Protean languages) based on a theory of Structured Operational Semantics (SOS) allows the definition of new operations that preserve basic semantic properties, without being overly extensive. A BDD-based model checker was parameterized by a SOS-defined specification language. This model checker successfully verified the specification written in a Protean language. He also presented a refined solution to the Dining Philosopher’s problem using this method.

Frank Stomp of AT&T Bell Laboratories presented “A Correctness Proof of a Cache Coherence Protocol” (coauthored by Amy Felty, AT&T). He outlined an experience in proving that a program satisfies its specification written in Linear Temporal Logic according to Scaleable Coherent Interface, a new IEEE standard for specifying communication between multiprocessors in a shared memory model. He asserted that this approach allows a transparent formulation of properties and structuring of their proofs.

Faron Moller of Uppsala University, Sweden, presented his view on the future of the computing industry. He strongly believes that education is the key to success; just as software engineers should learn about application domains, the domain experts should learn about software engineering. His paper, “The Specification of an Asynchronous Router,” provided a basic understanding of formal design tools such as Milner’s Calculus of Communicating Systems, the modal mu-calculus and Edinburgh Concurrency Workbench. He also reported on a meeting of the Association for Computing Machinery, Inc. (ACM) that discussed strategic directions of the ACM where many of the groups focused on formal methods.

## 10. Software Safety

John Knight, University of Virginia, moderated the Software Safety session. Jon Reese of the University of Washington presented “Safety Analysis Tools for Requirements Specifications” (coauthored by Vivek Ratan, Kurt Partridge and Nancy Leveson, University of Washington). He described safety analysis tools that have been developed for a state-based requirements specification language called Requirements State Machine Language. These tools include multiview interface, backward and forward execution of the specifications, fault tree generation, verification of correctness of specifications, and other safety analysis techniques. These tools were applied to analyze Automated Highway System.

A. M. Dearden of the University of York, U.K., described the “Impact and the Design of the Human Machine Interface” (coauthored by M. D. Harrison, University of York). He stressed the importance of Human-Machine Interface (HMI) and the relationship between operator actions and system hazard conditions. The impact of operator actions could be quantitatively assessed, and this assessment could be used to measure the merits of a particular HMI design. Such assessments could be used for further improving the HMI design and hence the safety of the system.

Reginald Meeson of the Institute for Defense Analysis presented “Object-Oriented—No Panacea for Safety.” His experience with embedded computer software systems showed that “object-oriented” does not necessarily imply safety. He noted certain pitfalls to watch out for while following certain object-oriented development techniques. In systems that were observed, these pitfalls led to a dangerous lack of visibility constraints and poorly designed concurrency that were both difficult to capture using the usual verification techniques.

## 11. Computer Security

John McLean, NRL, moderated the session on computer security. Tomas Olafson, Chalmers University of Technology, Sweden, presented the paper “An Empirical Model of the Security Intrusion Process” (coauthored by Erland Jonsson, Chalmers University). Starting with the hypothesis that the more effort it takes to break in, the more secure a system is, he asked, “How do you measure the effort?” Real data is not available. To address this challenge, students were given the task to breach the system. This effort provided data to determine the statistical distribution of the breaches. For this experiment, the breaches over time appeared to have an exponential distribution with an expected value of four hours.

Andrew Moore, NRL, discussed “Increasing Assurance with Literate Programming Techniques” (coauthored by Charles Payne, Secure Computing Corporation). He stated it is difficult to construct both persuasive and cost-effective arguments. Techniques exist that support high assurance. For example, architectures with criticality partitions of critical functions can be rigorously analyzed. What is needed is a framework for arguing about criticality functions using formal and informal techniques. Improved methods are also needed. Literate Programming techniques offer an approach. Lessons learned are provided in the paper.

Todd Fine, Secure Computing Corporation, presented “A Framework for Composition,” describing how analysis of complex systems requires the use of a “divide-and-conquer” approach to specification and verification. Building upon existing theories for specification composition, he described a technique for constructing more complex specifications by building upon simpler specifications.

“An Analysis of a Secure System Based on Trusted Components,” coauthored by Ulf Lindqvist, Tomas Olovsson, and Erland Jonsson (Chalmers University of Technology, Sweden), described a practical security analysis of a beta implementation of a commercial system based on existing trusted hardware components. The analysis was performed by means of document reviews, interviews and some practical tests with the intention of finding and listing potential vulnerabilities for the design team. The problems were to a high degree non-technical, reflecting organizational and management issues and human insufficiencies.

## 12. Testing

The testing session was moderated by Karen Ferraiolo of Arca Systems. Jie Pan of PRC, Inc. presented her Master’s Thesis for George Mason University: “Detecting Equivalent Mutants and the Feasible Path Problem” (coauthor Jeff Offutt, George Mason University). She discussed a mutation testing technique, where mutants of a program are created and the test results of the mutants are compared with the test results of the original. She also discussed detecting equivalent mutants, constraint-based testing, and recognizing infeasible constraints. To illustrate, she gave an example of a program which returns the minimum value of a list of numbers and showed how it can be mutated and analyzed.

Mark Blackburn of the Software Productivity Consortium, presented “T-VEC: A Tool for Developing Critical Systems” (coauthored by Robert Busser, Motorola) which described the T-VEC (Test Vector Generator) tool, including the T-VEC environment and the T-VEC development process. He explained automated test vector generation with the tool. He also described test selection strategy, problem domain test selection, and computing expected outputs. To illustrate, he gave an example of the second certification release of the MD90 aircraft Electrical Power System Variable Speed Constant Frequency System, a software package written in Ada on which the T-VEC was used to analyze the software.

Anup Ghosh of RST Corporation discussed “Defining an Adaptive Software Security Metric from a Dynamic Software Failure Tolerance Measure” (coauthored by J. Voas, G. McGraw, and F. Charron, RST Corporation and K. Miller, University of Illinois). He discussed his view of the history and current status of computer security, and the need to apply a specific type of analysis to security software prior to release. He discussed fault classes, test-case generation, fault injection, intrusion monitoring, and the development of relative security metrics such as Minimum Time To Intrusion. He gave an example of a Medical Scanner System.

## 13. COMPASS ’97

COMPASS ’97 will be held June 16–20, 1997, at NIST in Gaithersburg, Maryland. Paper abstracts must be RECEIVED by October 21, 1996, and the full paper submitted by November 8, 1996. For information about COMPASS ’97 or to obtain proceedings of COMPASS ’96, please contact Dolores Wallace, NIST, Building 820, Room 517, Gaithersburg, MD 20899-0001; telephone (301) 975-3340, fax (301) 926-3696, e-mail: dwallace@nist.gov, or see the web page at http://hissa.ncsl.nist.gov/compass/.

